# Identification and Comparative Analysis of MicroRNAs Associated with Low-N Tolerance in Rice Genotypes

**DOI:** 10.1371/journal.pone.0050261

**Published:** 2012-12-05

**Authors:** Lata Nischal, Mohd Mohsin, Ishrat Khan, Hemant Kardam, Asha Wadhwa, Yash Pal Abrol, Muhammad Iqbal, Altaf Ahmad

**Affiliations:** Molecular Ecology Laboratory, Department of Botany, Faculty of Science, Jamia Hamdard (Hamdard University), New Delhi, India; Centro de Investigación y de Estudios Avanzados del IPN, Mexico

## Abstract

**Background:**

Nitrogen [N] is a critical limiting nutrient for plants and has to be exogenously supplied to many crops, to achieve high yield with significant economic and environmental costs, specifically for rice. Development of low-input nitrogen sustainable crop is necessary for sustainable agriculture. Identification of regulatory elements associated with low-N tolerance is imperative for formulating innovative approaches for developing low-N tolerant crop plants, using gene manipulation. MicroRNAs (miRNAs) are known to play crucial roles in the modulation of gene expression in plants under various environmental conditions.

**Methodology/Principal Findings:**

MiRNAs associated with low-N tolerance have not been identified so far. In this study, we investigated microarray-based miRNA expression in low-N tolerant and low-N sensitive rice genotypes under low N condition. Expressions of 32 miRNAs differed significantly in the two genotypes. Of these 32 miRNAs, expressions of nine miRNAs were further validated experimentally in leaves as well as in roots. Of these differentially expressed miRNAs, six miRNAs (miR156, miR164, miR528, miR820, miR821 and miR1318) were reported in leaves and four (miR164, miR167, miR168 and miR528) in roots. Target genes of all the 32 miRNAs were predicted, which encode transcription factors, and proteins associated with metabolic processes or stress responses. Expression levels of some of the corresponding miRNA targets were analysed and found to be significantly higher in low N-tolerant genotype than low-N sensitive genotype. These findings suggested that miRNAs played an important role in low-N tolerance in rice.

**Conclusions/Significance:**

Genome-wide differences in expression of miRNA in low N-tolerant and low N-sensitive rice genotypes were reported. This provides a platform for selection as well as manipulation of genotypes for better N utilization efficiency.

## Introduction

Nitrogen (N) is a major factor, limiting crop productivity in field conditions [1], [2]. The global use of N fertilizer increased several-folds in the last 5 decades in order to augment crop productivity, because most of the high yielding varieties of the major crops developed during this period have high demands of N. However, plants consume less than half of the fertilizers applied [3]. Unused fertilizer N causes global warming through nitrous oxide emissions [4] and pollution of water by nitrate leaching [5]. Nitrous oxide is 300 times more potent than CO_2_ in its global warming impact [6]. Moreover, fertilizer application has now become the major cost in crop production, and significantly diminishes the income of farmers. Thus, developing crops that are less dependent on heavy application of N fertilizers is essential for the sustainability of agriculture. Technically, crop varieties that obtain N nutrient from soils with low N concentration (high uptake efficiency), and optimize the use of the absorbed N nutrient for production (high utilization efficiency) are required. There is a considerable challenge ahead in finding effective genetic and other innovations to develop such crop plants, to help minimize the use of N fertilizer without slowing improvements in crop productivity [7]. The failure to improve Nitrogen-Use-Efficiency (NUE) in transgenic plants by over-expressing individual enzymes of nitrate and ammonia assimilatory pathways has strengthened the view that metabolic flux through these pathways may be controlled by regulatory switches outside these pathways [8]. Although genomics and transcriptomics approaches characterized the global plant responses to nitrogen limitation [9]–[17], the regulatory mechanisms involved are still unknown.

Recently, microRNAs (miRNAs), a large family of endogenous small RNAs, were reported to play crucial roles in the modulation of gene expression [18], [19]. Functional studies showed that miRNAs are implicated in most of the essential physiological processes in plants, including organ development, signal transduction, responses under abiotic stress and biotic stress [20]–[23], and nutrition stresses [24]. MiR395 and miR399 have been found to mend regulatory processes under sulphur and phosphorus limitations, respectively [25]–[29]. Nitrogen responsive miRNAs have been investigated recently in *Arabidopsis* and maize [30], [31]. However, the miRNAs associated with the low-N tolerance have not been investigated so far. Investigation of natural mechanisms of low-N tolerance is an important strategy for understanding the biological basis of response to low-N condition. In our earlier study, we identified low-N tolerant (IC-547557) and low-N sensitive (Vivek Dhan) rice (*Oryza sativa*) genotypes [32]. In order to gain insight into the role of miRNAs in tolerance to low-N condition, we used these two rice genotypes for identifying miRNA associated with low-N tolerance. Rice is a major cereal crop and produces food for many populations of the world. The proportion of N fertilizer lost in rice fields is higher than that of other cereal crops [33]. In addition, N fertilizer application is also a major economic cost for rice farmers, especially in developing countries. Thus, the development of rice genotypes that can optimize N in low-nitrogen soils is essential for sustainable agriculture [33]. Our comparative analysis of expression patterns of miRNAs in low-N tolerant and low-N sensitive rice genotypes under limited N condition indicates that some miRNAs possess different levels of expression in different genotypes, and may, therefore, be a correlating factor for different levels of low-N tolerance among genotypes.

## Materials and Methods

### Plant Culture and Treatment

In our previous study [32], we identified low-N tolerant (IC-547557) and low-N sensitive (Vivek Dhan) rice genotypes. The seeds of these genotypes were procured from National Bureau of Plant Genetic Resources, New Delhi, India. Both the genotypes were grown at very low-N level (0.01 mM N) and optimum-N level (0.1 mM N) hydroponically. Kronzucker et al. [34] have earlier used 0.1 mM N as optimum level of N for the growth of rice for 3–4 weeks. The seeds were surface sterilized with 2% sodium hypochlorite solution, and then washed thoroughly with distilled water. Seeds were germinated in the dark at 27°C on blotting paper saturated with deionized water. After 96 h, seedlings were transferred to a hydroponic system placed in a growth chamber with a day/night regime of 16/8 h, PPFD of 200 µmol m^−2^ s^−1^ at plant level, temperature of 22°C in the dark, 26°C in the light and with a relative humidity of 65%. Seedlings were grown using the nutrient medium containing 2 mM K_2_SO_4_, 2 mM MgSO_4_, 1 mM CaCl_2_, 0.3 mM NaH_2_PO_4_, 40 µM Fe-EDTA, 9 µM MnCl_2_, 25 µM Na_2_MoO_4_, 20 µM H_3_BO_3_, 1.5 µM ZnSO_4_, and 1.5 µM CuSO_4_. Nitrogen in the form of NH_4_NO_3_ was maintained as 0.01 mM (low-N condition) and 0.1 mM (optimum-N condition). The pH of the nutrient solutions was adjusted to 6.0 and the solutions were changed every three days. All hydroponic solutions were continuously aerated by an electric pump. Twenty one-day-old plants of Vivek Dhan genotype showed symptoms of N-deficiency, when grown at 0.01 mM N. There was no symptom of N-deficiency in IC-547557 at this N level. Physiological status of the both the genotypes was measured in terms of biomass accumulation, leaf chlorophyll and nitrogen concentrations and expression level of N-responsive marker gene, *OsNRT2 (AB008519.1*; nitrate transporter). For biomass analysis, root and shoot were separated, dried at 65°C for 72 h and then weighed. Total nitrogen concentration was determined using a CHNOS analyzer (ELEMENTAR VARIO EL III, Germany). Chlorophyll concentration in leaves was measured by the method of Hiscox and Israelstem [35]. Expression level of *OsNRT2* was evaluated through quantitative real time PCR. Twenty-one day old plants were, therefore, taken for all the studies, grown at 0.01 mM N (low-N condition).

### RNA Isolation and Quantitation

Leaf and root tissue samples were immediately freezed in liquid nitrogen and used for RNA isolation. RNA was isolated using TRIZOL® reagent (Invitrogen, USA) with additional isopropanol overnight precipitation at −20°C. Concentration of isolated RNA was determined using iT™ RiboGreen RNA assay Kit (Invitrogen, USA). To minimise the loss of low molecular weight fraction during isolation procedure, total RNA was used for microarray and qRT-PCR analysis. The quality and integrity of total RNA was checked on 1.2% agarose gel saturated with formaldehyde, and that of small RNA on 15% PAGE saturated with 1.0 M urea. Samples were dissolved in RNAse-DNAse-free water and stored at –80°C until analysis.

### Microarray of miRNA and Data Analysis

For this procedure two biological replicates (whole plant) of genotype IC-547557 and Vivek Dhan was used to compare the expression of miRNAs under low-N (0.01 mM) condition. 500 ng of total RNA from plant tissue was used to label 3'-end of RNA molecules with Biotin-labelled DNA® using Flash Tag® Biotin HSR RNA Labelling kit (Genisphere, LLC, USA) for Affymetrix® Gene chip miRNA Arrays. For polyA tailing, 500 ng of total RNA was mixed with 2 µl of spiked control oligos, 1.5 µl of 10 × reaction buffers, 1.5 µl of 25 mM MnCl_2_, 1.0 µl of diluted ATP mix, and 1.0 µl of polyA polymerase enzyme per reaction, and then incubated at 37°C for 15 min. For biotin labelling, 15 µl of tailed RNA was mixed with Flash tag biotin HSR ligation mix and T_4_ DNA ligase.

Before proceeding for array, confirmation of labelling was done through ELOSA QC assay (Enzyme Linked Oligosorbent Assay, Affymetrix). For array preparation (GeneChip miRNA_2.0 Array), 21.5 µl of biotin labelled samples were incubated with array hybridization cocktail which includes 2× hybridization buffer, 27.5% formamide, dimethyl sulphoxide (DMSO), 20× eukaryotic hybridization controls and control Oligonucleotide (from Gene Chip eukaryotic hybridization control kit). 100 µl of this cocktail was injected into arrays and incubated in hybridization oven at 48°C and 60 rpm for 16 h. This cocktail was washed and stained with Fluidics Station 450. Filled array was then scanned using Gene chip scanner 3000 7G.

Probe specific signal detection call was based on Wilcoxon Rank sum test of the miRNA probes compared to the distribution of signal from GC content. Filtering was done to remove unimportant and unreliable data. After scanning data summarization, normalization and quality control was done through *miRNA QC Tool* software version 1.1.1.0, available at www.affymatrix.com. Normalized data is log2 transformed value. To identify if a feature value is significantly greater than the background value, a two sample t-test was used to report p-value. Positive and significant p-value of probe was taken as 0.05 or less. The normalized data was analyzed according to p-values of the *t*–test. All microRNAs having p-value ≤0.05 were statistically analyzed with significance analysis of microarray (SAM) software (http://www-stat.stanford.edu/~tibs/SAM/). The selection of significantly expressed microRNAs was done on the basis of q<0.0001 and having fold change ≥2. Data of log transformed values are presented in Table S1.

### Clustering Analysis

Average linkage method was used for the hierarchical clustering algorithm. An upper diagonal similarity matrix is computed for any set of genes using similarity score of all pair of genes, which is displayed graphically by colours. To observe the expression variation of miRNAs among low-N tolerant and low-N sensitive rice genotypes, we prepared hierarchal clusters based on data after normalization and statistical analysis. To see the trends among families of miRNAs in low-N tolerant and low-N sensitive rice genotypes, miRNAs were arranged according to hierarchical clustering method of Seo and Shneiderman [36] using “Hierarchical clustering explorer version 3.0″ programme (http://www.cs.umd.edu/hcil/hce/).

### Prediction of Target Genes of miRNAs

Plant miRNAs complement their target mRNAs by perfect or near-perfect base pairing. Based on a sequence similarity search, a web-based computing system (miRU) (http://plantgrn.noble.org/psRNATarget/) [37] was used to predict target mRNAs for the miRNAs associated with low-N tolerance by mature miRNA sequences. The miRU program reports all potential sequences, with mismatches no more than specified for each mismatch type.

The minimal score among all 20-mers cannot exceed 3.0 with default parameters. The functions of target genes were obtained from preloaded transcript/genomic library of *Oryza sativa* MSU Rice genome annotation release 6.1. The predicted target genes were categorized according to their functions using AmiGO Version 1.8 (http://amigo.geneontology.org/cgi-bin/amigo/go.cgi) and GRAMENE. Predicted targets through miRU were given in Table S2.

### Microarray Data Formatting and Deposition

All microarray data discussed in this publication had been processed into MIAME compliant data and deposited in NCBI's Gene Expression Omnibus and are accessible through GEO Series accession number GSE38213(http://www.ncbi.nlm.nih.gov/geo/query/acc. cgi?acc = GSE38213).

### Real-time PCR of Mature miRNAs and their Target Genes

Quantitative real-time PCR (qRT-PCR) of mature miRNAs from leaves and roots was done using miR-specific stem-loop primers for reverse transcription approximately 50 nt in length (44–45 nucleotides common to stem and 6–5 nucleotide for loop structure were miRNA-specific) and subsequently amplified using miRNA specific forward primer and universal reverse primer following procedure of Chen et al. [38]. The stringent criteria were used to get primer specificity and efficiency and melting temperatures were kept in the range of 58–60°C. Primers were checked for self complementarities and BLAST was done against all known transcripts in *Oryza sativa* so as to minimise non-specific amplifications. Product lengths of stem-loop reverse transcribed mature miRNAs were in the range of 60–75 nt. Sequences of the qRT-PCR primers along with that of internal control are given in Table S3.

A total of 10 ng of total RNA was reverse transcribed using 1.0 µM SLRT Primer, 0.5 µl 10 mM dNTPs, 11.15 µl of nuclease free water, 4 µl of 5× first strand synthesis buffer, 2 µl of 0.1 M DTT**,** and 200 units/µl concentration of reverse transcriptase (Invitrogen, USA) under cycling condition of 30°C for 16 min and 60 cycles of 30 °C at 30 s, 42 °C for 30 s, 50 °C for 1 s and reaction terminated at 85 °C. Stem loop primers were purchased from Integrated DNA Technologies (IDT, USA) and primers for real time PCR analysis from Sigma-Aldrich (USA).

Real time PCR of selected miRNA and target mRNAs was carried out through SYBR green chemistry [39] on a real time thermal cycler (Light Cycler 480, Roche, USA). MiR159 was used as internal control for miRNA (since among the other analysed miRNAs the Ct change between root and leaves of the both the genotypes ≤0.5) and actin gene was as internal control used for mRNA. 1 µl of cDNA was used with cocktail containing 2 µl of 10× buffer, dNTPs, MgCl_2_, miR specific forward and stem loop complimentary universal reverse primer along with 1× concentration of SYBR GOLD fluorescent dye (Invitrogen, USA) in cycling condition of hot start at 94°C for 2 min, 40 cycles of denaturation at 94°C for 15 s, and annealing and extension at 60°C for 1 min. All reactions were run in duplicates of three biological replicates.

The ΔΔC_T_ method was used to determine the expression level differences among samples of IC-547557 and Vivek Dhan. For a given genotype at low-N treatment, ΔΔC_T_ = (C_TmiRNA-VIVEK DHAN_ - C_TmiRNA159_) – (C_TmiRNA-IC-547557_ - C_TmiRNA159_) based on equation 9 of ΔΔC_T_ method [40]. Standard errors and standard deviation were calculated from replicates and significance was measured through Students’ *t*–test at the level of p≤0.05.

## Results

**Figure 1:**
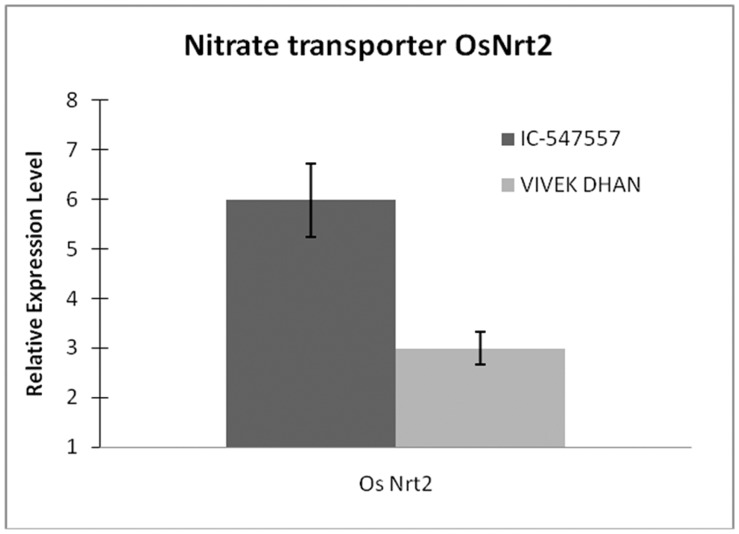
Relative expression level of nitrate transporter gene, *OsNrt2*, in 21-day-old plants of IC-547557 and Vivek Dhan at 0.1 mM **N.** Values are mean of duplicates of three biological replicates. SD is shown by bar.

Rice genotypes (IC-547557 and Vivek Dhan) showed apparent differences after 21 days of sowing in low-N condition. While IC-547557 genotype continued to grow relatively well, Vivek Dhan genotype of rice displayed symptoms of N-deficiency such as chlorosis of older leaves. The biomass of the IC-547557 genotype of rice was slightly higher at 0.1 mM N (optimum N-level) than at 0.01 mM N (low-N level), demonstrating the low-N tolerance property of this genotype. Contrary to this, the Vivek Dhan genotype of rice accumulated 2.4- fold lesser biomass at 0.01 mM N than at 0.1 mM N, demonstrating that low-N condition substantially limited the growth of this genotype. At low-N condition, the concentration of total nitrogen and chlorophyll were significantly higher in IC-547557 than in Vivek Dhan (Table 1). The physiological status of these rice genotypes was further validated through the expression of the marker gene, *OsNrt2*. Expression level of marker gene was significantly higher in IC-547557 than in Vivek Dhan (Fig. 1). For studying the role of miRNAs in low-N tolerance, the IC-547557 and Vivek Dhan genotypes of rice were, therefore, grown hydroponically in a growth chamber at 0.01 mM N for twenty-one days.

**Table 1:** Biomass accumulation and physiological status of IC-547557 (low-N tolerant) and Vivek Dhan (low-N sensitive) genotypes of rice, grown at low (0.01 mM) and optimum (0.1 mM) levels of N.

	IC-547557		VIVEK DHAN	
	0.01 mM N	0.1 mM N	0.01 mM N	0.1 mM N
Shoot biomass (mg)	18.2±0.34	19.1±0.21 ^ns^	11.1±0.12	25.1±0.76*
Root biomass (mg)	7.6±0.05	8.4±0.32 ^ns^	5.17±0.23	14.2±0.34*
Plant biomass (mg)	25.8±0.28	27.5±0.23 ^ns^	16.2±0.18	39.3±0.67*
Root/shoot ratio	0.41±0.22	0.44±0.21 ^ns^	0.46±0.15	0.56±0.53*
Chlorophyll content (mg g^−1^ FW)	1.58±0.08	1.64±0.03 ^ns^	0.89±0.02	1.54±0.12*
Total nitrogen content (mg g^−1^ DW)	34.7±0.09	36.8±0.04 ^ns^	24.8±0.02	37.4±0.04*

The results are averages ± SE of three biological replicates. Significance (p≤0.05) of the changes found between low-N and optimum-N conditions was checked with Student’s *t-*test, and is indicated by asterisk.

Comparative expression patterns of miRNAs in low-N tolerant (IC-547557) and low-N sensitive (Vivek Dhan) rice genotypes under N-limited condition (0.01 mM) were investigated using miRNA microarrays (Affymetrix GeneChip® miRNA 2.0 Arrays system). Microarray analysis of miRNA expression in low-N tolerant and low-N sensitive rice genotypes revealed that thirty two miRNAs belonging to 15 families were differentially expressed (Table 2). These miRNAs are miR156k, miR156l, miR164c, miR164d, miR164e, miR166l, miR167a, miR167b, miR167c, miR167d, miR167e, miR167f, miR167h, miR167i, miR167j, miR168a, miR169a, miR169b, miR528, miR535, miR820a, miR820b, miR820c, miR821a, miR821b, miR821c, miR1318, miR1432, miR1846a, miR1846e, miR1876 and miR2123, belonging to miR156, miR164, miR166, miR167, miR168, miR169, miR528, miR535, miR820, miR821, miR1318, miR1432, miR1846, miR1876, and miR2123 families. It was found that miRNA members of the same family had similar expression profiles, probably owing to highly homologous sequences that were difficult to distinguish even by using hybridization-based methods. Thus, variations of ≥2.0 fold were considered significant for study. Expression of all the reported miRNAs was significantly lower (2–8 folds) in IC-547557 than in Vivek Dhan (Table 2)**.** Family of miR167 showed 2–3 fold lesser expression level in this genotype compared to Vivek Dhan. Major changes were observed in miR528 and miR821b, in which 7.55-fold and 6.48-fold lesser expression were reported, respectively in IC-547557, when compared with the expression in Vivek Dhan.

**Table 2:** Differentially expressed miRNA in IC-547557 and Vivek Dhan genotypes of rice under low-N condition.

miRNA family	miR-ID	Fold increase (+)/decrease (−)	Sequence of miRNA in *Oryza sativa*
miR156	miR156k	−3.09	UGACAGAAGAGAGAGAGCACA
	miR156l	−2.47	CGACAGAAGAGAGUGAGCAUA
miR164	miR164c	−3.22	UGGAGAAGCAGGGUACGUGCA
	miR164d	−2.69	UGGAGAAGCAGGGCACGUGCU
	miR164e	−2.44	UGGAGAAGCAGGGCACGUGAG
miR166	miR166l	−2.70	UCGGACCAGGCUUCAAUCCCU
miR167	miR167a	−2.49	UGAAGCUGCCAGCAUGAUCUA
	miR167b	−3.06	UGAAGCUGCCAGCAUGAUCUA
	miR167c	−2.35	UGAAGCUGCCAGCAUGAUCUA
	miR167d	−2.59	UGAAGCUGCCAGCAUGAUCUG
	miR167e	−2.45	UGAAGCUGCCAGCAUGAUCUG
	miR167f	−2.27	U±GAAGCUGCCAGCAUGAUCUG
	miR167h	−2.91	UGAAGCUGCCAGCAUGAUCUG
	miR167i	−2.48	UGAAGCUGCCAGCAUGAUCUG
miR168	miR168a	−3.35	UCGCUUGGUGCAGAUCGGGAC
miR169	miR169a	−3.37	CAGCCAAGGAUGACUUGCCGA
	miR169b	−2.22	CAGCCAAGGAUGACUUGCCGG
miR528	miR528	−7.55	UGGAAGGGGCAUGCAGAGGAG
miR535	miR535	−2.29	UGACAACGAGAGAGAGCACGC
miR820	miR820a	−4.97	UCGGCCUCGUGGAUGGACCAG
	miR820b	−5.28	UCGGCCUCGUGGAUGGACCAG
	miR820c	−4.29	UCGGCCUCGUGGAUGGACCAG
miR821	miR821a	−5.20	AAGUCAUCAACAAAAAAGUUGAA
	miR821b	−6.48	AAGUCAUCAACAAAAAAGUUGAAU
	miR821c	−3.66	AAGUCAUCAACAAAAAAGUUGAAU
miR1318	miR1318	−3.21	UCAGGAGAGAUGACACCGAC
miR1432	miR1432	−3.02	AUCAGGAGAGAUGACACCGAC
miR1846	miR1846a-5p	−3.18	AGUGAGGAGGCCGGGGCCGCU
	miR1846e	−5.52	AGUGAGGAGGCCGGGGCCGCU
miR1876	miR1876	−2.74	AUAAGUGGGUUUGUGGGCUGGCCC
miR2123	miR2123c	−2.63	UAAAAAGUCAACGGUGUCAAAC

The values indicate the fold-change decrease/increase in the miRNA expression in IC-547557 over Vivek Dhan. Significance Analysis of Microarrays (SAM) and a criterion of fold change >2 and q value <0.001 was used to report the variation in expression.

**Figure 2:**
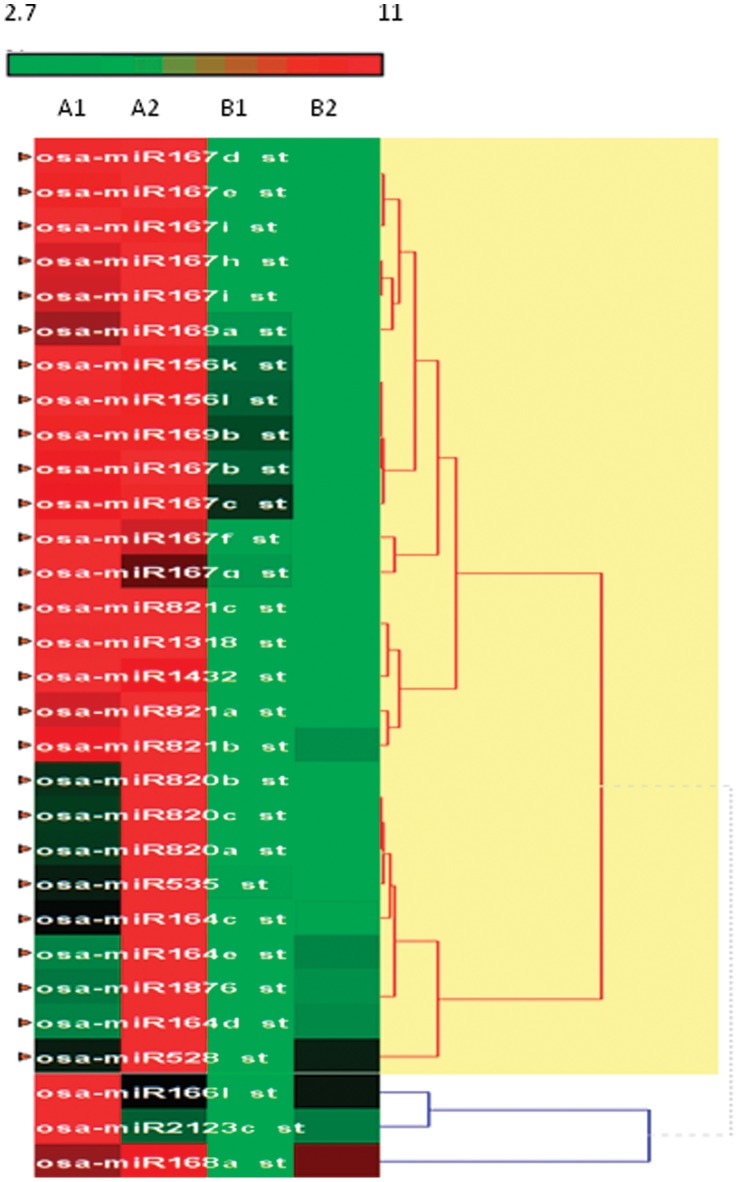
Heat map and cluster view of known microRNAs from *Oryza sativa.* Biological replicates A1 and A2 represent Vivek Dhan and B1 and B2 represent IC-547557. The scale bar represents scale of relative expression levels of microRNAs. Heat map was generated for all 32 variably expressed microRNAs having statistically positive and significant P value ≤0.05. The hierarchal clustering algorithm used is based on the average linkage method which was developed for clustering correlation matrix. These represents fluorescence ratios, cell with log ratio of 0 are coloured black increasing positive log ratios represented as red intensity and increasing negative log ratios with green intensity.

On the basis of the expression pattern, the identified 32 miRNAs were organized into 2 major clusters in one group and a separate cluster showing miR166l, miR2123c and miR168a (Fig. 2). MiRNAs with high degree of sequence similarity or miRNAs from the same family clustered in immediate vicinity of each other. Interestingly, cluster analysis revealed a stronger tendency of genes having common functions to congregate in one cluster.

### Validation of miRNA in Leaves and Roots of Rice Genotypes through Real Time PCR

**Figure 3:**
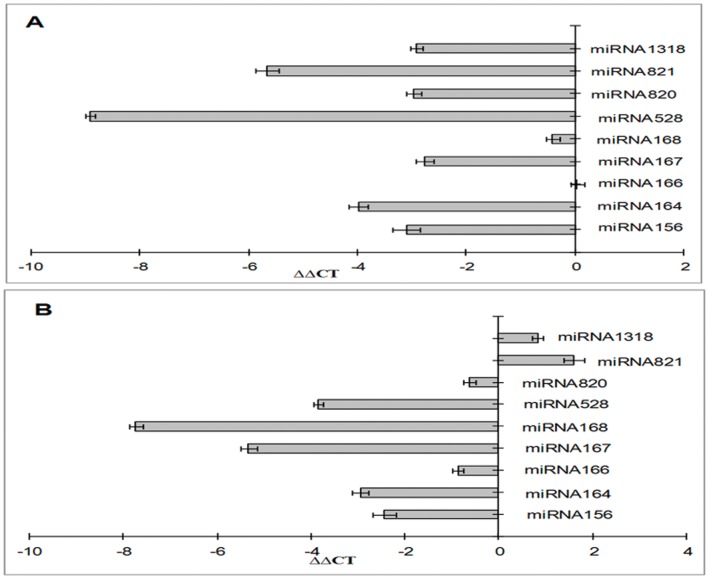
ΔΔC_T_ values of low-N tolerant miRNAs in leaves (A) and roots (B) of IC-547557 over Vivek Dhan. Significantly varied miRNAs were selected on basis of **ΔΔ**C_T_ ≥3 or ≥ −3. Values are means of duplicates of three biological replicates. SE is shown by bar. **ΔΔ**C_T_ = (**Δ**C_T –VIVEKDHAN-_(**Δ** C_T- IC-547557_ ) & **Δ**C_T_ = C_TmiR_ -C_T_miR159.

**Figure 4:**
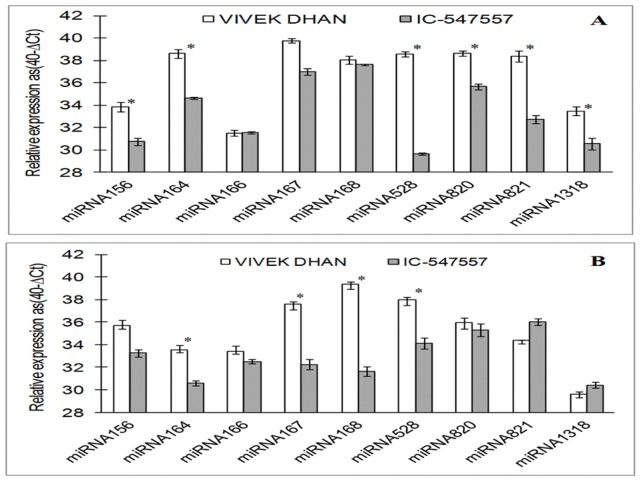
Quantitative real-time PCR analysis of miRNA levels in leaves (A) and roots (B) of low-N tolerant (IC-547557) and low-N sensitive (Vivek Dhan) genotypes of rice under low-N condition. The expression levels of miRNAs were normalized to the level of miR159 (doesn’t vary significantly between two genotype at jugged condition). Expression levels are given on a logarithmic scale expressed as 40-**Δ**C_T_, where **Δ**C_T_ is the difference in qRT-PCR threshold cycle number of the respective miRNA and the reference miRNA159; therefore, 40 equals the expression level of miR159 (the number 40 was chosen because the PCR run stops after 40 cycles). Significantly varied miRNAs were selected on basis of **ΔΔ**C_T_ ≥3 or ≥ −3. The results are averages ± SE of duplicates of three biological replicate. Significance of the changes between IC-547557 and Vivek Dhan under N-limitation was checked with Student’s *t*-test at the level of p≤0.05. miRNAs with significant expression difference between the two genotypes is shown as asterisk.

To validate the microarray results of genome wide analysis, nine differentially expressed miRNAs from miR156, miR164, miR166, miR167, miR168, miR528, miR820, miR821 and miR1318 families were selected for the validation of their expression levels. We designed nine stem-loop primers for nine variably expressed microRNAs, e.g. for miR820a-c, we used single RT-primer for cDNA synthesis. These miRNAs were verified by stem loop coupled real time PCR on mature miRNAs. Significant expression pattern of miRNAs was measured on the basis of change in normalized cyclic threshold ≥3 ( ΔΔC_T_≥±3) in the leaves (Fig. 3A) and roots (Fig. 3B) of low-N tolerant and low-N sensitive rice genotypes. Comparative expression levels of miRNAs in IC-547557 and Vivek Dhan are shown in Figure 4 and Table S4. Six miRNAs (miR156, miR164, miR528, miR820, miR821 and miR1318) showed differential expression in the leaves, when comparison was made between Vivek Dhan and IC-547557 genotypes of rice (Fig. 4A). Similarly, differential expression of four miRNAs (miR164, miR167, miR168 and miR528) was found in roots (Fig. 4B). The expression levels of these miRNAs were lesser in IC-547557 than in Vivek Dhan**.** The analysis of the expression of the mature miRNAs showed the consistency of most of the results between the microarray and the qRT-PCR where all the miRNAs were potentially showed lower expression in low-N tolerant genotype.

To work out the roles of the identified miRNAs in low-N tolerance genotype of rice (IC-547557), potential miRNA targets were identified by using psRNATarget server (an updated version of miRU) and *Oryza sativa* MSU Rice genome annotation release 6.1. Based on the criteria for maximum expectation equal to 3 as per default set up, these 32 miRNAs were found to target a total of 157 genes and most of these encode genes for transcription factors, enzymes of various metabolic pathways as well as structural proteins (Table S2). On the basis of importance of these genes in biological, cellular and molecular processes, we classified the genes into seven broad domains, involving carbohydrate/lipid metabolism, nitrogen metabolism, protein/peptide degradation, secondary metabolism, stress enzymes/cell rescue, defence and virulence, kinases/receptors/signalling, molecule/transporters, organ development and structural proteins (Table 3).

**Table 3:** Functional categorization of target genes on the basis of AmiGO description.

Accession number	Protein description	miR-ID
**Carbohydrate/lipid metabolisms/Energy metabolism**		
LOC_Os05g42110.1	Allyl alcohol dehydrogenase, putative expressed	miR168
LOC_Os08g04460.1,.2	NADPH dependent FMN reductase domain	miR168
LOC_Os08g04310.1,	Plastocyanin like domain containing protein, putative	miR528
LOC_0s12g16350.1	Enoyl-CoA hydratase/isomerase, putative	miR821
LOC_Os08g36910.1,2,3	Alfa –amylase precursor, putative	miR1423
LOC_Os02g18870.1	GDSL-like lipase/acyl hydrolase, putative	miR1846
LOC_Os010g33960.1,2	START-domain containing protein	miR166
**Nitrogen metabolism, amino acid metabolism and protein/peptide degradation**		
LOC_Os06g05760.1	Ubiquitin family protein, putative	miR164
LOC_Os03g48180.1,2	PTR2,Peptide transporter, putative	miR168
LOC_Os12g42400.2,	Ribosomal family, protein	miR169
LOC_Os01g44330.1	Laccase precursor, putative	miR528
LOC_Os03g574040.1,2	Clathrin adaptor complex small chain domain	miR156
**Secondary metabolism**		
LOC_Os06g03830.1,2	Retinol dehydrogenase, putative	miR167
LOC_Os02g21520.1	Chalcone isomerase, putative	miR168
LOC_Os11g13650.1	Cellulose synthase, putative	miR820
LOC_Os04g35590.1	Thioesterase family protein, putative	miR1318
**Stress enzymes/Cell rescue, defense and virulence**		
LOC_Os07g29820.1	NBS-LRR disease resistance protein, putative	miR167
LOC_Os10g17790.1	Remorin C terminal domain containing, putative	miR168
LOC_Os08g44770.1,2	Cu/Zn SOD, putative	miR528
LOC_Os01g03620.1	Multi copper oxidase, putative	miR528
LOC_Os02g01680.1	Macrophage migration inhibition protein, putative	miR2123
LOC_Os04g57200.2	Heavy metal transport and detoxification, protein	miR164
LOC_Os06g06050.1	OsFBL27 F-Box LRR	miR528
LOC_Os06g37150.1	L-ascorbate oxidase precursor, putative	miR528
**Kinases/receptors/signaling molecule/transporters**		
LOC_Os04g41540.1	OsCML22 Calmodulin related calcium sensor protein	miR164
LOC_Os02g43430.1	Kinase domain	miR156
LOC_Os01g63290.2	Transporter major facilitator family, putative	miR167
LOC_Os11g44860.1	Cysteine rich receptor like kinase, putative	miR168
LOC_Os03g59770.1,2	EF Hand, putative	miR1318
LOC_Os04g51610.1	Ca transporting ATPase, putative	miR1318
LOC_Os01g45830.1	Sulphate transporter, putative	miR2123
**Organ development**		
LOC_Os11g30370.1	OsSPL-SBP box gene family member	miR156
LOC_Os02g58490.1	PINHEAD protein	miR168
LOC_Os03g07880.1,2,3	Nuclear transcription factor Y subunit, putative	miR169
LOC_Os06g23650.1	NAM domain containing proteins, putative	miR164
LOC_11g03310.	NAM domain containing proteins, putative	miR820
**Structural proteins**		
LOC_Os09g33800.1	Arabinogalactan putative, expressed	miR528
LOC_Os04g47580.1	Cyclin putative, expressed	miR535
LOC_Os02g38340.1	Actin putative, expressed	miR821

**Figure 5:**
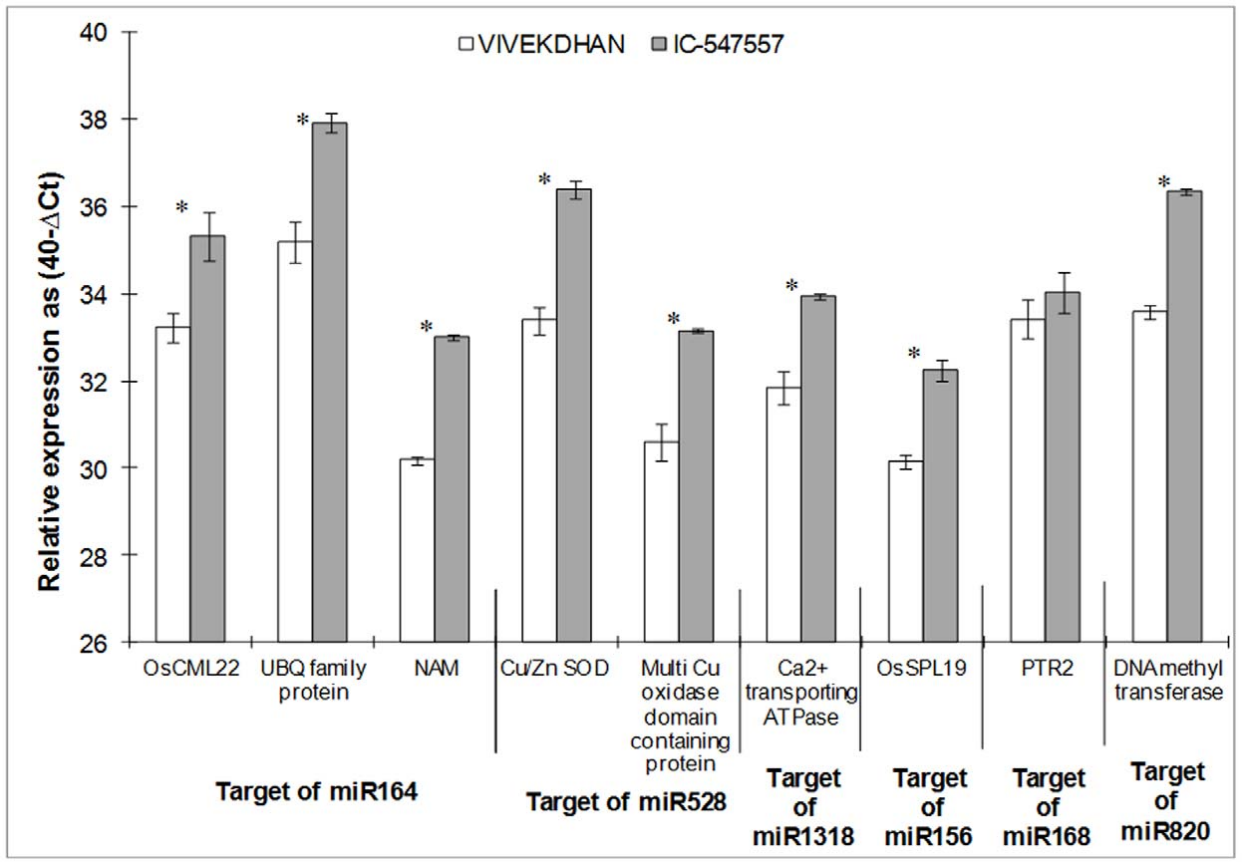
Expression profiling analysis of several target genes in the leaves of low-N tolerant (IC-547557) and low-N sensitive (Vivek Dhan) rice genotypes under low-N condition. The expression levels of miRNAs were normalized to the level of actin. Expression levels are given on a logarithmic scale expressed as 40−**Δ**C_T_, where **Δ**C_T_ is the difference in qRT-PCR threshold cycle number of the respective miRNA and the reference actin gene; therefore, 40 equals the expression level of actin gene (the number 40 was chosen because the PCR run stops after 40 cycles). The results are averages ± SE of duplicates of three biological replicate. Significance of the changes between IC-547557 and Vivek Dhan under N-limitation was checked with Student’s *t*-test at the level of p≤0.05. The significant expression difference between the two genotypes is shown as asterisk.

**Figure 6:**
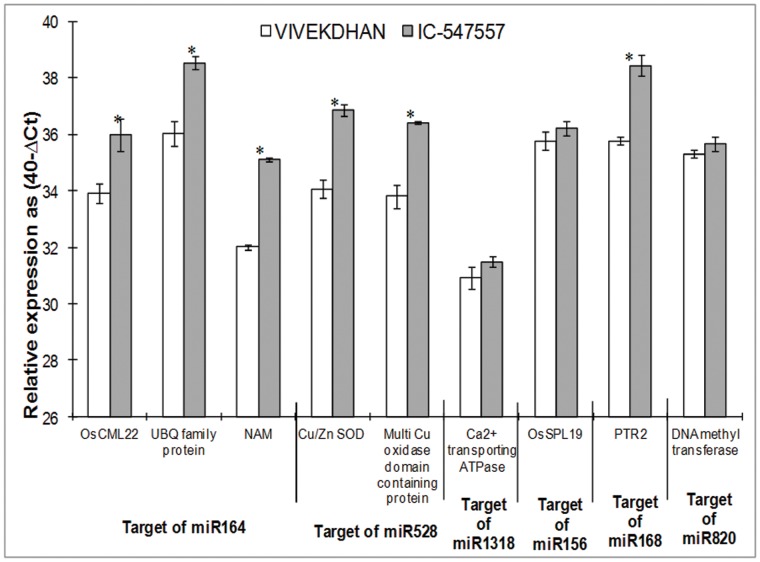
Expression profiling analysis of several target genes in the roots of low-N tolerant (IC-547557) and low-N sensitive (Vivek Dhan) rice genotypes under low-N condition. The expression levels of miRNAs were normalized to the level of actin. Expression levels are given on a logarithmic scale expressed as 40−ΔC_T_, where ΔC_T_ is the difference in qRT-PCR threshold cycle number of the respective miRNA and the reference actin gene; therefore, 40 equals the expression level of actin gene (the number 40 was chosen because the PCR run stops after 40 cycles). The results are averages ± SE of duplicates of three biological replicate. Significance of the changes between IC-547557 and Vivek Dhan under N-limitation was checked with Student’s *t*-test at the level of p≤0.05. The significant expression difference between the two genotypes is shown as asterisk.

In order to find possible miRNA/target gene modules that are differentially regulated in leaves and roots of IC-547557 and Vivek Dhan under low-N condition, the expression profiles of some predicted target genes were examined by qRT-PCR (Figs. 5 and 6). Since an analysis of target genes of miR169 and miR167 family had already been evaluated in earlier studies [17], [30], we selected few target genes from miR156, miR164, miR168, miR528, miR820 and miR1318 families to observe the expression pattern in low-N tolerant (IC-547557) and low-N sensitive (Vivek Dhan) rice genotypes under nitrogen limited condition. Fold-change expression of targets of identified miRNAs is given in Table 4. Expression levels of target genes of miR164 i.e., OsCML22-Calmodulin-related calcium sensor protein, ubiquitin family protein and NAM, were higher in leaves (4.22, 6.68 and 7.18-folds, respectively) and roots (4.23, 5.65, 8.46-folds, respectively) of IC-547557 genotype, when compared with expression in Vivek Dhan genotype. Similarly, the expression of miR168b target PTR2 (a peptide transporter protein) was ≥5-folds higher in roots of IC-547557 than in Vivek Dhan. Cu/Zn SOD (Cu/Zn superoxide dismutase) and multicopper oxidase, the targets of miR528, were significantly higher (≥6.0) in leaves and roots of IC-547557. Similarly, expression of genes of Ca^2+^ transporting ATPase (miR1318 target), Os-SPL19-SBP box gene family member (miR156 target) and DNA methyltransferase (miR820 target) were higher in leaves of IC-547557 when compared with Vivek Dhan. Expressions of Ca^2+^ transporting ATPase gene and DNA methyltransferase gene in roots were not significantly differed between the two genotypes (Figs. 5 and 6).

**Table 4:** Fold-change expression value of predicted targets of differentially expressed microRNAs in leaves and roots of IC-547557 over Vivek Dhan genotypes of rice.

miR-ID	Targets	Fold increase (+)/decrease (−)	
		Leaf	Root
miR156k/l	OsSPL-SBP-box gene family member	4.34	5.42
miR164c/d/e	No apical meristem protein (NAM)	7.18	8.46
	Calmodulin related Ca^2+^ sensor protein	4.22	4.23
	Ubiquitin family protein putative	6.68	5.65
miR168a	Peptide transporter PTR2, putative expressed	1.98	6.30
miR528	Copper/zinc superoxide dismutase, putative expressed	7.98	6.83
	Multicopper oxidase domain containing protein	5.83	6.02
miR820a,b,c	DNA methyltransferase protein, putative expressed	6.68	1.80
miR1318	Calcium-transporting ATPase membrane-type, putative expressed	4.33	1.46

The expression level is expressed as the mean of relative fold changes of duplicates of triplicate biological replicates. Fold change was measured as 2-**ΔΔ**CT where **ΔΔ**CT = (CT-target-VIVEK DHAN - CT-actin) -(CT-target-IC-547557 -CT-actin).

## Discussion

Regulation of gene expression through sequence-specific interaction between miRNAs and their target mRNAs offers an accurate and inheritable mechanism for plants to respond to environment stimuli [24] **.** Since development of low-N tolerant rice varieties is imperative for sustainable agriculture, extensive efforts are needed to discover genetic elements and mechanisms of low-N tolerance. miRNAs are emerging as potential regulators of gene expression and their proven promising role in regulating nutrient related gene network [25]–[31] may hold the key to understand genetic variation in biodiversity that can help in selection as well as manipulation to get high performing genotype under low N-fertilizing condition. However, miRNA associated with low-N tolerance are still to be identified. Here, we compared expression levels of miRNA in low-N tolerant and low-N sensitive rice genotypes under N-limited condition using Affymetrix GeneChip microarray and identified differential expression of miRNAs between IC-547557 and Vivek Dhan genotypes of rice. Affymetrix GeneChip_2.0 array contains 15,644 probe sets from 131 species including all 491 known rice miRNA probes from miRBase release 15. Using this highly sensitive and wide platform 32 differentially expressed miRNAs were identified in the two genotypes under low N-condition. These miRNAs belong to miR156, miR164, miR166, miR167, miR168, miR169, miR528, miR535, miR820, miR821, miR1318, miR1432, miR1846, miR1876, and miR2123 families. It has been generally assumed that miRNA expression inversely correlated with target transcript accumulation. The expression levels of some of the target genes have been validated by qRT-PCR. Interestingly, as per array, expressions of these miRNAs were 2–8 folds lower in low-N tolerant genotype than the low-N sensitive genotype, suggesting that expressions of their target genes were turned on in response to N deficiency in the former genotype. This list of miRNAs includes some miRNA families that were known to be associated with N-response in other plant species, indicating that they are involved in conserved N response pathways. For example, miR164, miR167, miR168 and miR169 showed differential expression patterns under nitrogen limitation in maize and *Arabidopsis*, [31], [41]. Some miRNAs (miR166l, miR535, miR820a-c, miR1432, miR1846, miR1876, miR2123 and miR1318) were reported for the first time in this study to be varied under N-limitation, suggesting that they may be involved in low-N tolerance pathways and functions. Validation of expression level of nine miRNAs was carried out in leaves and roots of IC-547557 and Vivek Dhan genotypes through real-time quantitative PCR.

Predicted target genes for all the differentially expressed miRNAs of rice genotypes encode proteins of diverse function; most of them being transcription factors. According to the characteristics of the predicted targets and their functions, the low N-responsive miRNAs had a strong propensity to function in plant development, signal transduction, and abiotic stress responses. For example, HD-Zip transcription factors targeted by miR166 were involved in shoot meristem initiation, leaf polarity establishment, and lateral root development [42], [43]. The HD-Zip transcription factors in *Arabidopsis* were also reported to be responsive to water deficiency, osmotic stress, and exogenous ABA treatment [44]. MiR166 was down-regulated by gibberellins (GA) in rice, followed by the elevated expression level of HD-Zip [45]. MiR156 is among the most conserved microRNAs in plants. Its target genes, SQUAMOSA promoter binding like (SPL) transcription factors, have been reported in *Arabidopsis*, maize, rice and wheat [46]–[48]. SPL’s are involved in the regulation of development during vegetative phase change [49]. Low level of expression of miR156 promotes adult phase development [49], [50]. OsSPL14/IPA1, one of miR156 target, has been reported as the regulator of panicle size [51], [52]. miR156-Overexpressing plants reported to have down-regulated expression of *CYP724* and *CYP90* (genes involved in brassinosteroid synthesis) and resulted in dwarfness, leaf erect and reduced panicle size in rice [53], indicating that reprogramming of development is a crucial step for plants to cope with low-N condition. Over-expression of miR156 promotes over-accumulation of anthocyanin (through SPL9) [54] causing redness and yellowing of leaves, a symptom associated to N-deficiency in plants [55]. Expression of target gene of miR164, NAM (no apical meristem) proteins, was significantly higher (7.18-folds in leaves and 8.46-folds higher in roots) in IC-547557 compared to Vivek Dhan under low-N condition. NAM proteins are involved in shoot apical meristem formation and auxin mediated lateral root development. NAC1, one of its targets, mediates auxin induced lateral root development. Transgenic *osaxr* (auxin insensitive mutants) have higher level of miR164 and defective in lateral roots development [56]. Moreover, QTL encoding a NAC is involved in nutrient remobilization from grain to leaves during senescence in wheat [57].

In comparison to Vivek Dhan, a significantly higher expression level of calmodulin related calcium sensor proteins, OsCML22 (predicted target gene of miR164) and Ca^2+^ transporting ATPases (predicted target gene of miR1318) in IC-547557 under low-N condition, suggested the involvement of a regulatory cascade of downstream metabolic processes under low N condition in rice genotype. Expression level of ubiquitin family proteins (predicted target of miR164) was higher in IC-547557 than in Vivek Dhan. This protein is involved in protein degradation. Ubiquitin ligases (*ATL31* and *ATL6*) target 14-3-3 proteins and regulate the C/N response via UPS (ubiquitin proteosome)-mediated degradation [58], thus are involved in a regulatory mechanism for primary carbon and nitrogen metabolism. Studies with NLA (nitrogen limitation adaptation) mutant *Arabidopsis* line showed that NLA is associated with UBC8, a member of *Arabidopsis* E2 proteins. These studies also indicated that nitrogen limited condition affected the switching over of anthocyanin biosynthesis to lignin biosynthesis pathway in NLA mutant, confirming the re-programming of the secondary metabolite pathways [59].

Expressions of all members of miR167 (a–j) were significantly lower in IC-547557 than in Vivek Dhan under low N condition. Earlier studies have reported that miR167a family regulates root pericycle cells of *Arabidopsis* through affecting ARF8, an auxin responsive transcription factor [17]. In rice, most of miR167 target genes encode ion transporter proteins, signaling kinases and one dehydrogenase, suggesting substantial effect of low N stress on cellular ion balance. Serine/threonine kinases are proteins, regulating metabolism through phosphorylation and dephosphorylation activity in changing state of enzymes and metabolites of carbon and nitrogen metabolism [60]. Their expressions were significantly up-regulated in rice root cells under nitrogen limitation [13].

Expressions of miR169a and miR169b were 3.37 and 2.22-folds lower, respectively in IC-547557 in comparison to Vivek Dhan. Variable expression pattern of miR169 family under N limitation was also reported earlier in roots and leaves of *Arabidopsis* [41], rapeseed [30] and maize [31]. NFY- A family transcription factor (nuclear factor Y, subunit A), the target of the miR169, has been reported to be down-regulated under N deprivation [41]. These transcription factors are involved in the expression of proteins of oxidative stresses [22]. Repression of miR169 resulted in tolerance to drought stress and thus may mediate low-N stress generated osmotic imbalance [61], [62].

Comparatively lesser expression of miR528 and higher expression level of its predicted target genes (superoxide dismutase and multicopper oxidase) in the roots and leaves of IC-547557 under low N-condition indicate that antioxidative defence system is also involved in conferring tolerance to low-N condition in IC-547557. Strong repression of miR528 was also reported in maize under N limitation [31], supporting its relation in low-N adaptive response. Induction of antioxidative defence mechanisms under N-limitation has been reported earlier [63].

Some of the targets of miR820 family (miR820a, miR820b and miR820c) are the genes encoding DNA methyltransferase. The miR820 direct methylation around its target site, *Os03g02010,* and thereby regulate its function through methylation [64] the same gene was more expressed in IC-547557 than in Vivek Dhan. Regulation of genotypic variability in the salt tolerance has been reported through variable methylation of genes in salt tolerant and salt sensitive rice genotypes. It was suggested that this response may be incorporated in genotypes with natural selection under stress [65]. In rice, miR168 family is represented by miR168a and miR168b. Differential expression of miR168a in roots tissue was found in both the genotypes. The targets of miR168a, the *PINHEAD* proteins (*ARGONAUTE* family), are important catalyst involving in the regulation of transcripts through maintaining miRNA abundance or shortage [66], [67]. A transgenic *AGO1* resistant to miR168 developed severe development defects, leading to death of plant [68]. The other predicted targets of miR168 are transporter proteins like major facilitator family, peptide transporters PTR2, metal cation transporter and sulphate transporter. Significantly higher expression of PTR2, the predicted target of miR168, was found in roots of IC-547557 compared to Vivek Dhan in our study. QTL analysis of genes in maize identified one of peptide transporter gene, *PTR2* (GRMZM2G065967), as significant genes involved in nitrogen metabolism and associated with nitrogen use efficiency under N limitation [69]. Induction of ion transporters under N limited condition suggested reprogramming of its uptake as well as translocation [70]–[72].

### Conclusions

In the present study, miRNA microarrays were used to profile the expression patterns of annotated miRNAs (miRBase release 17; (http://www.mirbase.org/cgi-bin/mirna_summary.pl?org=osa) in low-N tolerant and low-N sensitive rice genotypes under N limitation, leading to the identification of 32 miRNAs associated with low-N tolerance. Expressions of all the miRNAs were lower in low-N tolerant genotypes compared to low-N sensitive genotype. Target gene prediction and subsequent expression analysis of identified low-N tolerant miRNA provided further evidence for the potential involvement of these miRNAs in low-N tolerance mechanism. The data set of the identified low-N responsive miRNAs is potentially important for additional characterization of the molecular mechanisms underlying low-N tolerance in rice. However, further functional analysis of N-responsive miRNAs is required to confirm their role in low-N tolerance in plants. Our findings also demonstrate that expression patterns of miRNAs may vary extensively even between two genotypes of the same species, in response to external stimuli. Further characterization of the targets of the identified miRNAs will help understand the details of response and tolerance to N-deficiency in rice.

## Supporting Information

Table S1 **Expression profile for all the miRNAs analysed in IC-547557 and Vivek Dhan under low-N condition (0.01 mM) using Affymetrix Gene Chip _2.0 array platform.**
(XLS)

Table S2 **List of predicted target genes of all 32 variably expressed miRNAs.**
(XLS)

Table S3 **List of full sets of primers used to amplify miRNAs and mRNAs including primers for control.**
(DOC)

Table S4 **Complete list of comparative Ct values of nine miRNAs significantly varied (in leaves and roots) between IC-547557 and Vivek Dhan.**
(XLSX)
