# Supplementary material for: Identification and Comparative Analysis of MicroRNAs Associated with Low-N Tolerance in Rice Genotypes
Source: PLoS One. 2012 Dec 5;7(12):e50261. doi: 10.1371/journal.pone.0050261 (PMC3515565; doi:10.1371/journal.pone.0050261)
Supplement: Table S3 — List of full sets of primers used to amplify miRNAs and mRNAs including primers for control. (DOC) [file pone.0050261.s003.doc]

**Table 3**. Primer sequences of miRNA and some of their target genes analysed through Real Time PCR

| **Primer name** | **Description** | **Primer sequence** |
| --- | --- | --- |
| **Stem-loop primer for reverse transcription** | | |
| miR156-SLRT |  | 5’GTTGGCTCTGGTGCAGGGTCCGAGGTATTCGCACCAGAGCCAACGTGCTC 3’ |
| miR164-SLRT |  | 5’GTTGGCTCTGGTGCAGGGTCCGAGGTATTCGCACCAGAGCCAACGCTCGT 3’ |
| MiR166-SLRT |  | 5’GTTGGCTCTGGTGCAGGGTCCGAGGTATTCGCACCAGAGCCAACTTGGGT 3’ |
| MiR167-SLRT |  | 5’GTTGGCTCTGGTGCAGGGTCCGAGGTATTCGCACCAGAGCCAACCTTGTT 3’ |
| MiR168-SLRT |  | 5’GTTGGCTCTGGTGCAGGGTCCGAGGTATTCGCACCAGAGCCAACCCCTG 3’ |
| MiR528-SLRT |  | 5’GTTGGCTCTGGTGCAGGGTCCGAGGTATTCGCACCAGAGCCAACCTCCTC 3’ |
| MiR820-SLRT |  | 5’GTTGGCTCTGGTGCAGGGTCCGAGGTATTCGCACCAGAGCCAACCTGGCT 3’ |
| MiR821-SLRT |  | 5’GTTGGCTCTGGTGCAGGGTCCGAGGTATTCGCACCAGAGCCAACTTCTTT 3’ |
| MiR1318-SLRT |  | 5’GTTGGCTCTGGTGCAGGGTCCGAGGTATTCGCACCAGAGCCAACGCTG 3’ |
| **Primers for real time PCR of miRNAs** | | |
| Universal reverse primer |  | 5’GTGCAGGGTCCGAGGT 3’ |
| miR156F |  | 5’GGCGGCTGACAGAAGAGAGT 3’ |
| miR164F |  | 5’TGG AGA AGC AGG GTA CGT GCA 3’ |
| MiR166F |  | 5’GGC TCG GAC CAG GCT TCA GTA 3’ |
| MiR167F |  | 5’TGAAGCTGCCAGCATGATCTA 3’ |
| MiR168F |  | 5’TCG CTT GGT GCA GGT CGG CTA 3’ |
| MiR528F |  | 5’GAG TGG AAG GGG CAT GCA TT 3’ |
| MiR820F |  | 5’TGC GTC GGC CTC GTG GAT G 3’ |
| MiR821F |  | 5’AAAGTCATCAACAAAAAAG 3’ |
| MiR1318F |  | 5’TGA TCA GGA GAG ATG ACA C 3’ |
| **Primers for real time PCR of target genes** | | |
| Os04g41540 | OsCML22 - Calmodulin-related calcium sensor protein, expressed | F-5’TTCTTCGACGACGACAACCAT 3’  R-5’TGCTGCTGCTGCTTCTTCTTGCT 3’ |
| Os06g05760 | ubiquitin family protein, putative | F-5’TCAAGCAGAAGGTGGAGAGTTGCT 3’  R-5’TGCGAGCCATCGAAGATGGGATAA 3’ |
| Os08g44770 | copper/zinc superoxide dismutase, putative, expressed | F-5’TGGCGATACTACGAATGGGTGCAT 3’  R-5’ACAGAATTTGGGCCACTCAGAGGA 3’ |
| Os01g03640 | multicopper oxidase domain containing protein, expressed | F-5’ACGTGGAAGAACGCGGTGAAGATT 3’  R-5’AGGATGTGGCAGTGGTAGACGAAT 3’ |
| Os04g51610 | calcium-transporting ATPase, plasma membrane-type, putative, expressed | F-5’AGTTGCTGACGGGTATGGCACTAT 3’  R-5’AGTACCTGGCCAGAAGGACAACAA 3’ |
| Os03g48180 | peptide transporter PTR2, putative, expressed | F-5’GCGGCATCAACAGCTTCTTCAACT 3’  R-5’CTGACGTTGCTCTGGACGTAGATGAT 3’ |
| Os11g303370 | OsSPL19 - SBP-box gene family member, expressed | F-5’TGGATGGTCATAACAGGCGTCGAA 3’  R-5’CAGCTATCGGAAATGTTTGCGGGT 3’ |
| 0s03g02010 | DNA methyltransferase protein, putative, expressed | F 5’CAAGCGCAAAGCTTACAGAGCGAA 3’  R 5’ AGCGACCTTATTCTTTCCGACCCA 3’ |
| Os10g051000 | *OsActin* | F 5’TACGAAGGTTATGCCCTTCCGCAT 3’  R 5’TCTTTGCAGTCTCAAGCTCCTGCT 3’ |
| miR159-SLRT |  | 5’GTTGGCTCTGGTGCAGGGTCCGAGGTATTCGCACCAGAGCCAACTGCTGC 3’ |
| miR159 |  | F 5’CCTACCTTGCATGCCCCAGGA 3’ |
| Nitrate transporter  OsNrt2 (AB008519.1) |  | F-5’ AAGGCGTAAACAATTCTGCGACCG 3’  R-5’ TCATCACCGTTTGCAACAAGGACG 3’ |
